# Remote Ischemic Preconditioning Reduces Perioperative Cardiac and Renal Events in Patients Undergoing Elective Coronary Intervention: A Meta-Analysis of 11 Randomized Trials

**DOI:** 10.1371/journal.pone.0115500

**Published:** 2014-12-31

**Authors:** Hanjun Pei, Yongjian Wu, Yingjie Wei, Yuejin Yang, Siyong Teng, Haitao Zhang

**Affiliations:** 1 Department of Cardiology, Fuwai Hospital, National Center for Cardiovascular Diseases, Chinese Academy of Medical Sciences and Peking Union Medical College, Beijing, 100037, China; 2 State Key Laboratory of Cardiovascular Disease, National Center for Cardiovascular Diseases, Chinese Academy of Medical Sciences and Peking Union Medical College, Beijing, 100037, China; Chinese Academy of Medical Sciences, China

## Abstract

**Background:**

Results from randomized controlled trials (RCT) concerning cardiac and renal effect of remote ischemic preconditioning(RIPC) in patients with stable coronary artery disease(CAD) are inconsistent. The aim of this study was to explore whether RIPC reduce cardiac and renal events after elective percutaneous coronary intervention (PCI).

**Methods and Results:**

RCTs with data on cardiac or renal effect of RIPC in PCI were searched from *Pubmed*, *EMBase*, and *Cochrane library* (up to July 2014). Meta-regression and subgroup analysis were performed to identify the potential sources of significant heterogeneity(*I* ^2^≥40%). Eleven RCTs enrolling a total of 1713 study subjects with stable CAD were selected. Compared with controls, RIPC significantly reduced perioperative incidence of myocardial infarction (MI) [odds ratio(OR)  = 0.68; 95% CI, 0.51 to 0.91; *P* = 0.01; *I^2^* = 41.0%] and contrast-induced acute kidney injury(AKI) (OR = 0.61; 95% CI, 0.38 to 0.98; *P* = 0.04; *I^2^* = 39.0%). Meta-regression and subgroup analyses confirmed that the major source of heterogeneity for the incidence of MI was male proportion (coefficient  = −0.049; *P* = 0.047; adjusted *R^2^* = 0.988; *P* = 0.02 for subgroup difference).

**Conclusions:**

The present meta-analysis of RCTs suggests that RIPC may offer cardiorenal protection by reducing the incidence of MI and AKI in patients undergoing elective PCI. Moreover, this effect on MI is more pronounced in male subjects. Future high-quality, large-scale clinical trials should focus on the long-term clinical effect of RIPC.

## Introduction

Procedure-related myocardial infarction(MI) [1], [2] and contrast-induced acute kidney injury(CI-AKI) [3], [4] following percutaneous coronary intervention (PCI) are two major complications in patients with stable coronary artery disease(CAD), and have been recognized as two important predictors of long-term adverse cardiovascular outcomes. The potential contributing mechanisms for these two phenomena include coronary microembolization, side branch occlusion, and reduced blood flow of the renal medulla [5], [6]. Although several drugs have been clinically used to increase cardiac and/or renal tolerance to the ischemic injury(such as statins [7] and N-acetylcysteine [8]), any single method may face challenge for the increasing aging and/or diabetic population. Thus, novel therapeutic strategies are required to provide benefits in patients undergoing PCI.

Remote ischemic preconditioning (RIPC) is an emerging approach whereby intermittent ischemic stimulus at an organ (mostly a limb) increases ischemic tolerance of a distant one to the subsequent ischemic insult. Accumulating evidence from various animal studies has supported the systemic protective potential offered by RIPC including heart and kidney [9], [10]. In humans, RIPC has also been shown to prevent reperfusion-induced endothelial dysfunction [11]. Based on these findings, growing interest in the translational potential of RIPC exists in the cardiovascular clinical practice [12].

Recently, randomized controlled trials (RCT) concerning cardiac [13]–[21] and/or renal [14], [18], [21]–[24] effect of remote ischemic preconditioning (RIPC) in patients with stable CAD are inconsistent. Hence, we conducted a meta-analysis to explore whether RIPC (compared with control) reduce cardiac and renal events after elective PCI.

## Materials and methods

### Searching Process

This meta-analysis was conducted according to the PRISMA (Preferred Reporting Items for Systematic reviews and Meta-Analyses) Statement [25]. A systematic search was performed in *PubMed*, *EMBase*, and *Cochrane Library* (up to July 2014), and scientific sessions (2010∼2013) of American Heart Association (AHA), American College of Cardiology(ACC), and European Society of Cardiology (ESC) using keywords “remote ischemic preconditioning”,“percutanenous coronary intervention”, “elective”, “cardiac”, “renal”, and“kidney”.

### Inclusion and Exclusion Criteria

Inclusion criteria were: <$>\raster="rg1"<$> prospective RCTs published in English; <$>\raster="rg2"<$> elective PCI. Studies involving patients with ST-segment elevation myocardial infarction were not included. Studies without reporting one of the two endpoints (incidence of myocardial infarction, and acute kidney injury) were also excluded.

### Study selection and quality assessment

Two investigators (Hanjun Pei and Yonggang Sui) independently reviewed all abstracts and included the full text in duplicate according to the described search strategy and criteria. Discussion was conducted for consensus in case of disagreement. Quality assessment was completed according to the Jadad scoring system: randomization; blinding; withdrawals and dropouts (a possible score between 0 and 5). Trials with a score of more than 3 were considered as high-quality studies [26].

### Data Extraction

Data extraction of study characteristic included trial design (year, country, PCI type, RIPC protocol, first cuff to balloon time and follow up) and patients characteristics [age, male, diabetes mellitus, hypertension, dyslipidemia, previous myocardial infarction (MI), smoke, number of vessels, baseline left ventricular ejection fraction(LVEF), baseline renal function and stenting technique, post-PCI thrombolysis in myocardial infarction (TIMI) grade, contrast volume, β-blockers, statins]. We tried to contact with the authors to ask for the related data, however, none of them responded.

### Postoperative Endpoints

The perioperative incidence of myocardial infarction (MI), and acute kidney injury (AKI) were the primary endpoints. The diagnostic criteria of MI was in accordance with the consensus of Joint ESC/ACCF/AHA/WHO(world health organization) Task Force for the universal definition of myocardial infarction in 2007 [27]: an elevation of troponin levels more than 3∼5 times the upper reference limit(URL). AKI was defined as follows: increase in serum creatinine (Cr ≥25% or ≥0.5 mg/dL) from baseline.

### Data synthesis and analysis

For dichotomous ones (reported with incidence), we calculated odds ratio (OR) with 95% confidence intervals (CIs). Random-effect model was used for analysis in case of significant heterogeneity among trials. In order to explore the potential influential factors affecting the reduction by RIPC in MI, we set “I^2^ value of 40%”as the cut-off value [28]. Publication bias was assessed by Begg's test and Egger's test. Sensitivity analysis was used to identify the influence of each included study on the overall estimate of MI and AKI. Meta-regression and subgroup analyses were performed to explore the potential sources of significant heterogeneity for postoperative endpoints (a P value of less than 0.05 was accepted). *P*<0.05 (2-sided) was considered to be statistically significant. All statistical analysis was performed in Stata (version 9.0; Stata Corporation, College Station, TX) and RevMan(version 5.0; Cochrane Collaboration, Oxford, UK).

## Results

### Study characteristics

**Figure 1:**
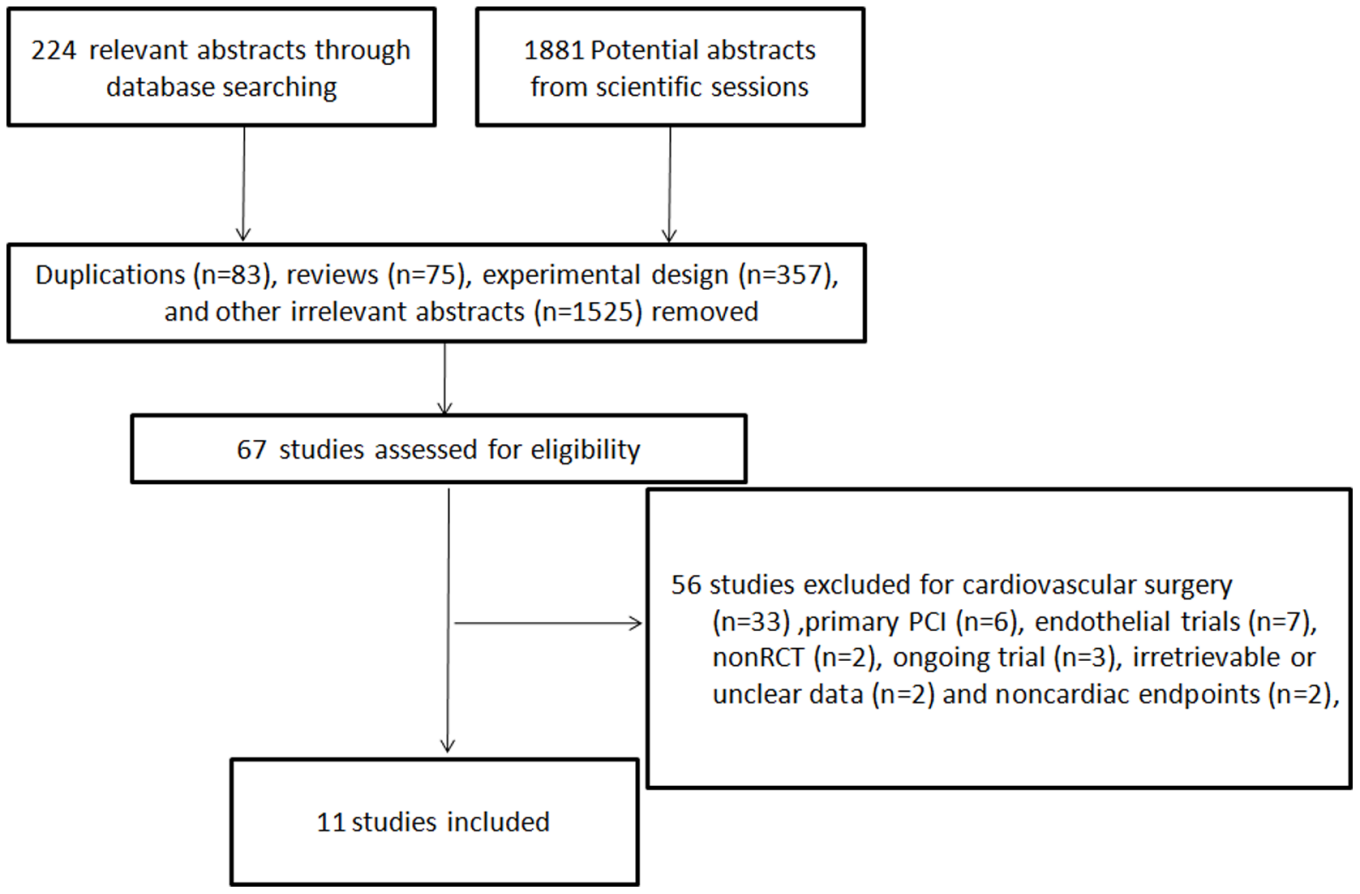
Searching process for the eligible studies. RCT, randomized controlled trial.

After 2040 abstracts were excluded from initial search due to duplications, reviews, experimental designs, and other irrelevant contents, sixty-seven potential studies were selected for detailed evaluation. Fifty-four studies were further excluded for the following reasons: cardiovascular surgery (n = 33), primary PCI(n = 6), endothelial trials (n = 7), nonRCT (n = 2), ongoing trial (n = 3), irretrievable or unclear data (n = 1) and noncardiorenal endpoints (n = 2). We also excluded one trial only reporting biomarkers of myocardial injury [13] and one trial reporting AKI without according to the presented definition(OR = 0.2; *P* = 0.05) [24]. Eleven trials [14]–[23], [29] with a total of 1713 patients ultimately met our criteria (Fig. 1). The ischemic protocol [cycles ×I/R(ischemia/reperfusion)] was 3∼4×5min/5min in eight studies [14], [17]–[19], [21]–[23], [29], 2×5min/5min in one [16], 3×3min/3min in one [15], and 1×5min/1min in one [20]. For the primary endpoints, the incidence of MI was reported in ten [14]–[22], [29], and the incidence of AKI in five [14], [18], [21]–[23]. We divided Lavi's trial [21] into two independent studies(expressed as Lavi I and Lavi II) according to the different conditioning protocols. Seven studies [14], [16], [18], [21]–[23], [29] had a Jadad score of more than 3. The study design and patient characteristics were summarized in Table 1 and 2.

**Table 1:** Summarized study design of included randomized trials.

Study	Country	PCI type	Pts. No. RIC vs Ctrl	RIC protocol			Control	First cuff to balloon time	Jadad score	Side Effect
				Cycles × I/R	Cuff pressure	Limb				
Hoole 2009^[14]^	UK	Elective	126 vs 125	3×5min/5min	200 mmHg	Upper arm	Placebo	96min	4	N.R
Prasad 2012^[15]^	US	Elective	40 vs 40	3×3min/3min	200 mmHg	Upper arm	Placebo	>18min	1	N.R
Ghaemian 2012^[16]^	UK	Elective	50 vs 50	2×5min/5min	> SBP	Lower arm	Placebo	65min	5	N.R
Er 2012^[23]^	Germany	Elective	26 vs 26	4×5min/5min	50 mmHg>SBP	Upper arm	Placebo	40∼85min	5	N.R
Ahmed 2013^[17]^	US	Elective	101 vs104	3×5min/5min	200 mmHg	Upper arm	Placebo	Several mins	1	N.R
Luo 2013^[18]^	China	Elective	126 vs 125	3×5min/5min	200 mmHg	Upper arm	Non-placebo	<120min	3	N.R
Xu 2013^[22]^	China	Elective	102 vs 98	3×5min/5min	200 mmHg	Upper arm	Non-placebo	30∼120 min	5	N.R
Chinchilla 2013^[29]^	Spain	Elective	118 vs 114	3×5min/5min	200 mmHg	Upper arm	Placebo	5min after PCI	5	3 with pain
Melo 2013^[19]^	Brazil	Elective	9 vs 20	3×5min/5min	200 mmHg	Upper arm	N.A	N.A	N.A	N.R
Lavi I 2014^[21]^	Canada	Elective	120 vs 120	3×5min/5min	200 mmHg or 50 mmHg>SBP	Upper arm	Placebo	Several mins after PCI	5	N.R
Lavi I 2014^[21]^	Canada	Elective	120 vs 120	3×5min/5min	200 mmHg or 50 mmHg>SBP	Thigh	Placebo	Several mins after PCI	5	N.R
Zografos 2014^[20]^	UK	Elective	47 vs 47	1×5min/1min	200 mmHg	Upper arm	Placebo	4min	2	N.R

Note: I/R, ischemia/reperfusion; SBP, systolic blood pressure; DBP, diastolic blood pressure; N.R, not report; RIC, remote ischemic conditioning; Ctrl, control.

**Table 2:** Summarized patient characteristics of included randomized trials.

Study	Age	Male(%)	Diabetes (%)	Pre-MI (%)	Baseline. LVEF(%)	HT (%)	Dyslipidemia(%)	Target Vessels ≥2	Baseline renal function	β-blockers(%)	Statins(%)
Hoole 2009^[14]^	62.5	78.2	21.8	55.4	50.2	51.5	N.A	16.8	N.A	79.2	95.0
Prasad 2012^[15]^	66.1	83.2	27.4	28.4	56.0	77.9	73.7	63.2	Normal	73.7	67.4
Ghaemian 2012^[16]^	59.9	47.5	36.3	8.8	N.A	48.8	73.8	1.3	Normal	81.3	76.3
Er 2012^[23]^	73.0	71.0	64.0	41.0	59.6	91.0	75.0	N.A	eGFR <60	82.0	N.A
Ahmed 2013^[17]^	54.1	86.6	51.7	N.A	N.A	63.8	66.4	N.A	N.A	N.A	72.5
Luo 2013^[18]^	59.3	76.1	27.8	21.5	64.0	65.9	N.A	27.8	eGFR = 100	82.4	N.A
Xu 2013^[22]^	69.0	68.0	100.0	23.0	63.7	63.5	N.A	N.A	Normal	80.0	100.0
Chinchilla 2013^[29]^	64.6	68.1	42.1	N.A	58.3	75.6	62.2	58.3	N.A	82.9	67.5
Melo 2013^[19]^	N.A	N.A	N.A	N.A	N.A	N.A	N.A	N.A	N.A	N.A	N.A
Lavi I 2014^[21]^	63.7	72.9	32.5	43.0	N.A	70.0	67.0	18.8	Normal	N.A	N.A
Lavi I 2014^[21]^	64.3	74.2	29.5	42.0	N.A	70.0	65.0	21.7	Normal	N.A	N.A
Zografos 2014^[20]^	60.5	88.0	19.0	20.0	56.4	82.0	71.5	24.5	eGFR = 88.4	82.0	96.0

### Effect of Remote Ischemic Preconditioning on the incidence of MI

**Figure 2:**
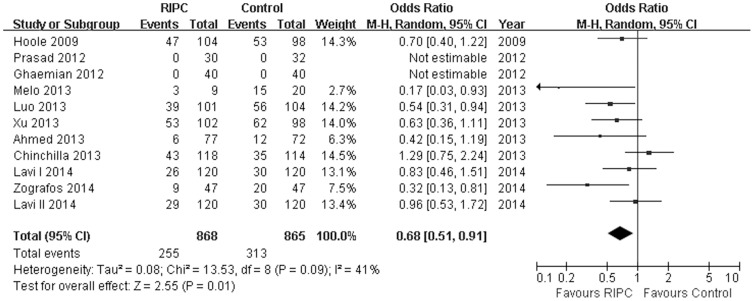
Forest plot for the incidence of myocardial infarction (MI). RIPC significantly decreased the risk of MI [odds ratio (OR)  = 0.68, *P* = 0.01].RIPC, remote ischemic preconditioning.

The MI was reported in 1613 study subjects, and the overall incidence was 33.35% (255/868 in RIPC group, 283/745 in control group). Perioperative incidence of MI was significantly reduced by RIPC (OR = 0.68; 95% CI, 0. 51 to 0.91; *P* = 0.01; *I^2^* = 41.0%; Fig. 2). No evidence of significant publication bias were observed for incidence of MI (*P* = 0.06, Begg's test; *P* = 0.13, Egger's test). Sensitivity analysis excluding each included study at one time revealed that the individual study was consisted with the direction and size of the overall MI reducing effect (All P≤0.03).

### Effect of Remote Ischemic Preconditioning on the incidence of AKI

**Figure 3:**
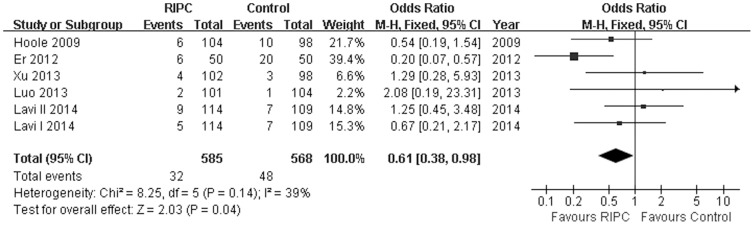
Forest plot for incidence of acute kidney injury (AKI). RIPC significantly prevented post-PCI AKI (OR = 0.61, *P* = 0.04). RIPC, remote ischemic preconditioning.

The AKI was reported in 1044 study subjects, and the overall incidence was 6.99% (32/585 in RIPC group, 41/459 in control group). A lowered risk of perioperative AKI was observed in the remote preconditioned patients(OR = 0.61; 95% CI, 0.38 to 0.98; *P* = 0.04; Fig. 3)with nonsignificant heterogeneity(*I^2^* = 39.0%). No evidence of significant publication bias were observed for the incidence of AKI (*P* = 0.57, Begg's test; *P* = 0.24, Egger's test). Sensitivity analysis excluding each included study at one time revealed that most individual study was consisted with the direction and size of the overall AKI reducing effect (All P≤0.05) with an exception of Er et al's (P = 0.65) or Hoole et al's (P = 0.09) study.

### Potential Sources of Significant Heterogeneity

**Figure 4:**
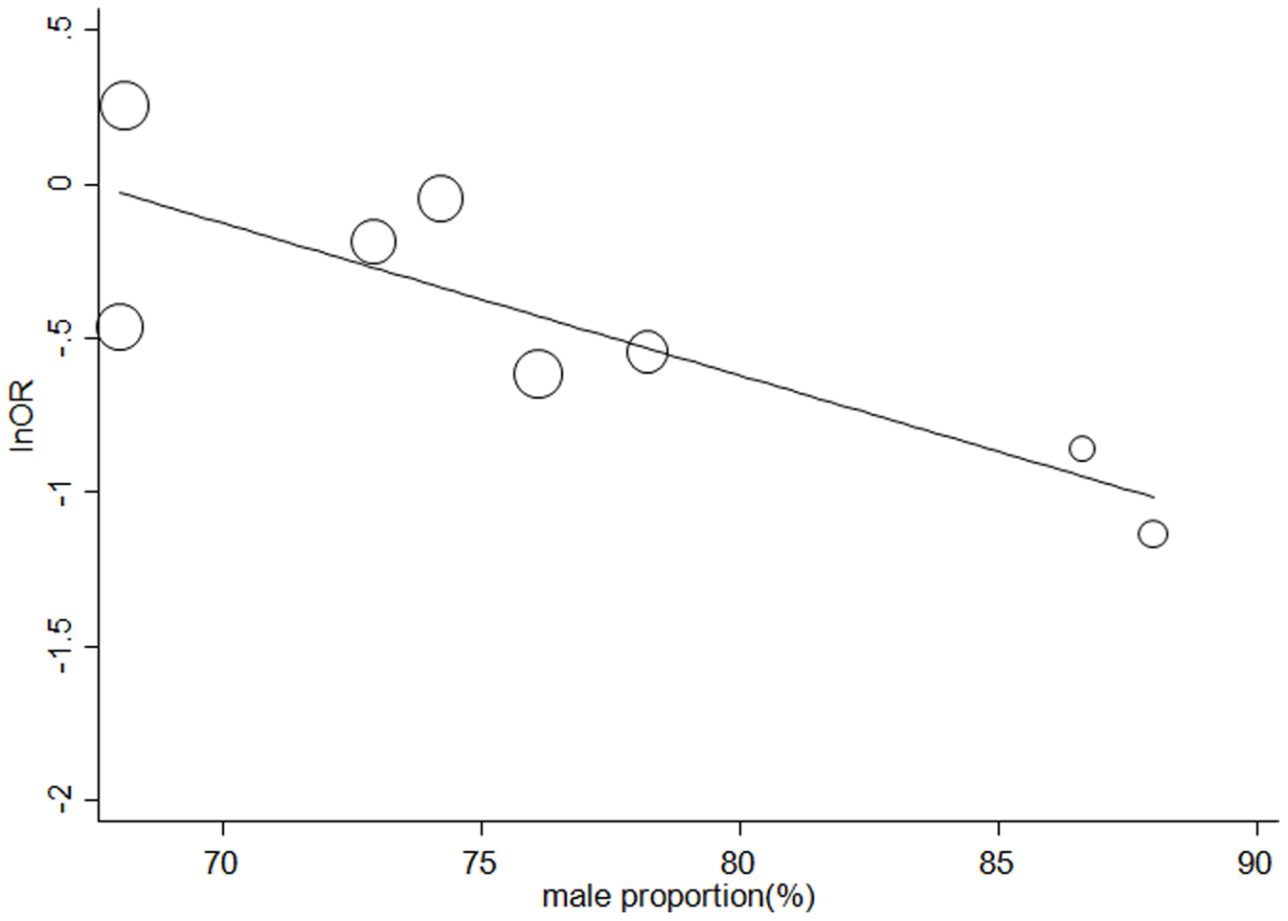
Meta-regression plots on the incidence of MI in PCI[Male proportion (%); coefficient  = −0.049; *P* = 0.047)].

Age, male proportion, diabetes proportion, history of MI proportion, baseline left ventricular ejection fraction, dyslipidemia proportion, hypertension proportion, target vessels ≥2 proportion, β-blockers usage, statins usage, and total conditioning time (cycles × duration of ischemic stimulus) were included in the random-effect univariate meta-regression analysis for the incidence of MI(ln transformation of OR) in PCI. As a result, the identified major source of heterogeneity was male proportion (coefficient  = −0.049; 95% CI, −0.0970 to −0.0008; *P* = 0.047; adjusted *R^2^* = 0.988) (Fig. 4). A subgroup with more than 75% of male subjects (OR = 0.54; 95% CI, 0.38 to 0.76) has a more profound effect size than that with less than 75% of male ones (OR = 0.90; 95% CI, 0.68 to 1.20)(*P* = 0.02 for subgroup difference; Table 3).

**Table 3:** Potential source of heterogeneity for the incidence of MI in PCI.

Variables	No. of Comparisons	Coeff./OR	95% CI	P Value	
***Univariate***		***Coeff.***			***Adjusted R^2^***
**Male(%)**	10	−0.049	−0.0970∼−0.0008	0.047	0.988
***Subgroup***		***OR***			***P_Difference_ Value***
	10	0.67	0.54∼−0.83	<0.00001	
**Male(%) ≥75%**	5	0.54	0.38∼0.76	0.0004	0.02
**Male(%) <75%**	5	0.90	0.68∼1.20	0.48	

Note: MI, myocardial infarction; Coeff., coefficient; OR, odds ratio; CI, Confidence Interval.

## Discussion

In the present systematic review and meta-analysis of 11 randomized trials enrolling 1746 patients undergoing elective PCI, we found that RIPC could offer cardiorenal protection by reducing the incidence of perioperative MI and AKI. Moreover, this effect on MI is more pronounced in male subjects. To our knowledge, this is the first meta analysis focusing on the effect of RIPC on cardiac and renal events in elective PCI.

Currently, the most widely used type of ischemic conditioning during cardiac intervention is ischemic postconditioning (IPoC) which performed by intermittently reinflating the stent balloon immediately after reperfusion (most within 1 min). IPoC has been demonstrated to reduce myocardial enzyme levels [30], [31], increase left ventricular function [30], limit the infarct size and edema [32], and may improve clinical outcomes [33], [34]. RIC is another endogenous approach with similar cardiac beneficial effect, as confirmed in our pooled analysis and Munk et al' study [35], [36]. On the other hand, the prevention of RIC for AKI in patients during cardiovascular procedure has been proposed in several clinical studies [23], [37], [38], indicating the systemic organ protective potential. Moreover, RIC was conducted by inflating an upper-arm blood pressure cuff in the most included trials, which makes it more applicable and harmfulless than IPoC in the clinical settings.

There has always been a concern whether cardioprotective effects of RIPC established in young and healthy animals could be translated into the clinical population with various co-morbidities and/or cofounders (such as gender) in clinical practice [12], [39]. Studies on infarct size reduction by ischemic postconditioning have shown to be gender-specific in animal models by other groups [40], [41]. Zhou et al [30] using meta regression analysis also found that reduction in post-PCI myocardial enzyme levels by IPoC is more evident in male patients than female ones. In our analysis, the male proportion ranged from 47.5% to 88.0%, and further pooled analysis suggested an increased effect size (ln transformation of OR of MI) by 0.49 per 10% increase in male proportion, which was further confirmed in the subgroup analysis. This first evidence from second analysis of RCTs indicating that cardioprotection by RIPC may be more pronounced in male subjects could provide some advice for the clinical usage of RIPC.

The optimal conditioning protocol(cycles ×I/R) for RIPC to elicit organ protection in human remains unknown. Only one laboratory study from Xin et al [42] found that 3∼4, but not 1∼2 cycles of 5-min/5-min RIPC could provide additive cardioprotection to local postconditioning, and the similar results were obtained in 4 cycles of 3-min/3-min or 1-min/1-min. In the current analysis, 8 of 11 studies used 3∼4 cycles of 5-min/5-min for conditioning. Prasad et al [15] did not find any protective effect of 3 cycles of 3-min RIPC on cardiac enzyme levels (cTnT or CK-MB), PCI-related myonecrosis rate, or MI occurrence. Two cycles of 5-min RIPC was also proved to reduce cardiac enzyme level and PCI-related myonecrosis rate by Ghaemian et al [16]. Moreover, one cycle of 5-min RIPC remained to be cardioprotective in Zografos's study [20]. Taken together, the current evidence suggest that 5-min ischemic stimulus for conditioning protocol in RIPC is essential. Future studies should verify whether increase in conditioning cycle of RIPC may result in enhanced organ protection in the clinical settings.

Several limitations should be pointed out in this study. Firstly, the potential influences of other co-morbidities (such as age, multivessel disease, diabetes, and dyslipidaemia) [30], [43], cardiovascular medications (such as adenosine, nitroglycerin, and statins) [44]–[46], and stenting techniques [30] may be underestimated for the lack of the individual patient data. Secondly, the baseline cardiac and renal function, pre-PCI TIMI grade, number of vessels, and contrast volume may be very important for the cardiorenal protection during PCI. However, we cannot explore their effect on RIPC-induced protection. Thirdly, the statistical power of our results may be inadequate because of the relative small number of the included studies and the enrolled subjects. Fourthly, no statistical significance in the Begg's and Egger's tests cannot rule out the potential impact of publication bias on our findings. Fifthly, other parameters for conditioning, such as the occlusion pressure and the limb, still need optimalization. Fifthly, in the included trials, the time window is quite wide-ranging in PCI (several ∼120 min). We tried to explore the potential effect by using meta-regression and subgroup analyses and failed to obtain an indicative finding(data not shown). Sixthly, the role of gender in the MI reduction by RIPC in elective PCI was indicated by meta regression and subgroup analyses. Whether it holds true in the individual subjects needs further investigation. Lastly, the long-term cardiorenal morbidity and mortality needs further evidence in future clinical trials.

## Conclusions

Available evidence from the present systematic review and meta-analysis suggests that RIPC may offer cardiorenal protection by reducing the incidence of MI and AKI in patients undergoing PCI. Moreover, this effect on MI is more pronounced in male subjects. Future high-quality, large-scale clinical trials should focus on the long-term clinical effect of RIPC.

## Supporting Information

S1 File (DOC)

S2 File (XLS)

S1 PRISMA Checklist (DOC)
