# Supplementary material for: Remote Ischemic Preconditioning Reduces Perioperative Cardiac and Renal Events in Patients Undergoing Elective Coronary Intervention: A Meta-Analysis of 11 Randomized Trials
Source: PLoS One. 2014 Dec 31;9(12):e115500. doi: 10.1371/journal.pone.0115500 (PMC4281209; doi:10.1371/journal.pone.0115500)
Supplement: S1 File — (DOC) [file pone.0115500.s001.doc]

Database Source: ***PubMed(n=10) or EMBASE(n=1)***

Ref.14.Hoole SP, Heck PM, Sharples L, Khan SN, Duehmke R, et al. (2009) Cardiac remote ischemic preconditioning in coronary stenting (CRISP Stent) study: a prospective, randomized control trial. Circulation 119: 820-827. ***PMID: 19188504***

Ref.15. Prasad A, Gossl M, Hoyt J, Lennon RJ, Polk L, et al. (2013) Remote ischemic preconditioning immediately before percutaneous coronary intervention does not impact myocardial necrosis, inflammatory response, and circulating endothelial progenitor cell counts: A single center randomized sham controlled trial. Catheter Cardiovasc Interv 81: 930-936. ***PMID: 22517646***

Ref.16. Ghaemian A, Nouraei SM, Abdollahian F, Naghshvar F, Giussani DA, et al. (2012) Remote ischemic preconditioning in percutaneous coronary revascularization: a double-blind randomized controlled clinical trial. Asian Cardiovasc Thorac Ann 20: 548-554. ***PMID: 23087298***

Ref.17. Ahmed RM, Mohamed EH, Ashraf M, Maithili S, Nabil F, et al. (2013) Effect of remote ischemic preconditioning on serum troponin T level following elective percutaneous coronary intervention. Catheter Cardiovasc Interv 82: E647-E653. ***PMID: 23404916***

Ref.18. Luo SJ, Zhou YJ, Shi DM, Ge HL, Wang JL, et al. (2013) Remote ischemic preconditioning reduces myocardial injury in patients undergoing coronary stent implantation. Can J Cardiol 29: 1084-1089. ***PMID: 23414904***

Ref.19. Melo RMV, Costa LMA, Uchida A, Oikawa FTC, Ribeiro HB, et al. (2013) Prevention of myocardial injury after percutaneous coronary interventions with remote ischemic preconditioning. A comparative analysis with biomarkers and cardiac magnetic resonance. European Society of Cardiology. Amsterdam, Netherlands. pp. 1009. [***http://www.embase.com/search/results?subaction=viewrecord&rid=2&page=1&L71261252***](http://www.embase.com/search/results?subaction=viewrecord&rid=2&page=1&L71261252)

Ref.20. Zografos TA, Katritsis GD, Tsiafoutis I, Bourboulis N, Katsivas A, et al. (2014) Effect of One-cycle Remote Ischemic Preconditioning to Reduce Myocardial Injury During Percutaneous Coronary Intervention. Am J Cardiol In press. ***PMID: 24793669***

Ref.21. Lavi S, D'Alfonso S, Diamantouros P, Camuglia A, Garg P, et al. (2014) Remote Ischemic Postconditioning During Percutaneous Coronary Interventions: Remote Ischemic Postconditioning-Percutaneous Coronary Intervention Randomized Trial. Circ Cardiovasc Interv In press. ***PMID: 24692535***

Ref.22. Xu X, Zhou Y, Luo S, Zhang W, Zhao Y, et al. (2013) Effect of Remote Ischemic Preconditioning in the Elderly Patients With Coronary Artery Disease With Diabetes Mellitus Undergoing Elective Drug-Eluting Stent Implantation. Angiology In press. ***PMID: 24163121***

Ref.23. Er F, Nia AM, Dopp H, Hellmich M, Dahlem KM, et al. (2012) Ischemic preconditioning for prevention of contrast medium-induced nephropathy: randomized pilot RenPro Trial (Renal Protection Trial). Circulation 126: 296-303. ***PMID: 22735306***

Ref.29. Carrasco-Chinchilla F, Munoz-Garcia AJ, Dominguez-Franco A, Millan-Vazquez G, Guerrero-Molina A, et al. (2013) Remote ischaemic postconditioning: does it protect against ischaemic damage in percutaneous coronary revascularisation? Randomised placebo-controlled clinical trial. Heart 99: 1431-1437. ***PMID: 23850844***
